# Model‐based analysis of episodic memory retrieval in healthy aging and mild cognitive impairment

**DOI:** 10.1002/alz.089119

**Published:** 2025-01-09

**Authors:** Paul F Hill, Joshua D Garren, Donette C Markham, Arne Ekstrom

**Affiliations:** ^1^ University of Arizona, Tucson, AZ USA

## Abstract

**Background:**

Episodic memory declines during healthy aging and is often reported as an early symptom of Alzheimer’s disease (AD). However, standardized assessments of memory performance are limited in their accuracy to predict progression of early‐stage AD pathology. The ‘all‐or‐none’ approach commonly used in neuropsychological assessment for quantifying memory performance might miss out on subtle variation in the fidelity or quality mnemonic representations retrieved from memory.

**Methods:**

Thirty‐five cognitively healthy older adults and nine older adults with MCI completed five study‐test cycles of a continuous report item‐location memory task. A subsample of 21 healthy older adults performed the task while undergoing high‐resolution fMRI. During the study phase, participants viewed trial‐unique objects located around the perimeter of an invisible circle. During the test phase, old and new objects were presented in the center of the screen and participants were instructed to recall the original location of each object using a continuous analogue response dial to indicate their response. Trial‐wise distance error between the remembered and true location of each object were fit with a probabilistic mixture model to estimate two distinct memory processes that are often conflated by categorical response memory paradigms: the ability to accurately retrieve prior details from memory (i.e., retrieval success), and the fidelity of details successfully retrieved from memory (i.e., memory precision).

**Results:**

Behavioral measures of retrieval accuracy and memory precision were significantly reduced in the MCI cohort relative to healthy older adults. Critically, we observed substantial individual differences in memory precision that were independent of retrieval success and performance on standardized assessments of verbal and visuospatial memory. Encoding‐related brain activity predictive of subsequent memory precision was evident in the left hippocampus along with robust bilateral effects in dorsomedial frontal and occipitotemporal cortices. Precision effects at retrieval were modest by comparison, yielding a single cluster in the left intraparietal sulcus.

**Conclusions:**

Precision‐based measures of episodic memory appear to capture subtle changes in cognitive function during early stages of AD progression that cannot be readily accounted for by standardized assessments of verbal and visuospatial memory.

